# Pathological Chemistry

**Published:** 1884-09

**Authors:** 


**Pathological Chemistry.** By CHARLES H. RALFE, M. D., F. R. C. P., Assistant Physician at the London Hospital.

These have been already favorably noticed in our columns, and we would again commend the entire series as by far the best of any works of this character which have been published.

